# Lessons Learned from West Nile Virus Infection:Vaccinations in Equines and Their Implications for One Health Approaches

**DOI:** 10.3390/v16050781

**Published:** 2024-05-14

**Authors:** Ahsan Naveed, Lianne G. Eertink, Dan Wang, Feng Li

**Affiliations:** Maxwell H. Gluck Equine Research Center, Department of Veterinary Science, University of Kentucky, Lexington, KY 40546, USA; ahsannaveed@uky.edu (A.N.); lianne.eertink@uky.edu (L.G.E.); dan.wang@uky.edu (D.W.)

**Keywords:** West Nile virus, equines, humans, vaccines, immunity, correlates of protection

## Abstract

Humans and equines are two dead-end hosts of the mosquito-borne West Nile virus (WNV) with similar susceptibility and pathogenesis. Since the introduction of WNV vaccines into equine populations of the United States of America (USA) in late 2002, there have been only sporadic cases of WNV infection in equines. These cases are generally attributed to unvaccinated and under-vaccinated equines. In contrast, due to the lack of a human WNV vaccine, WNV cases in humans have remained steadily high. An average of 115 deaths have been reported per year in the USA since the first reported case in 1999. Therefore, the characterization of protective immune responses to WNV and the identification of immune correlates of protection in vaccinated equines will provide new fundamental information about the successful development and evaluation of WNV vaccines in humans. This review discusses the comparative epidemiology, transmission, susceptibility to infection and disease, clinical manifestation and pathogenesis, and immune responses of WNV in humans and equines. Furthermore, prophylactic and therapeutic strategies that are currently available and under development are described. In addition, the successful vaccination of equines against WNV and the potential lessons for human vaccine development are discussed.

## 1. Introduction

**Figure 1:**
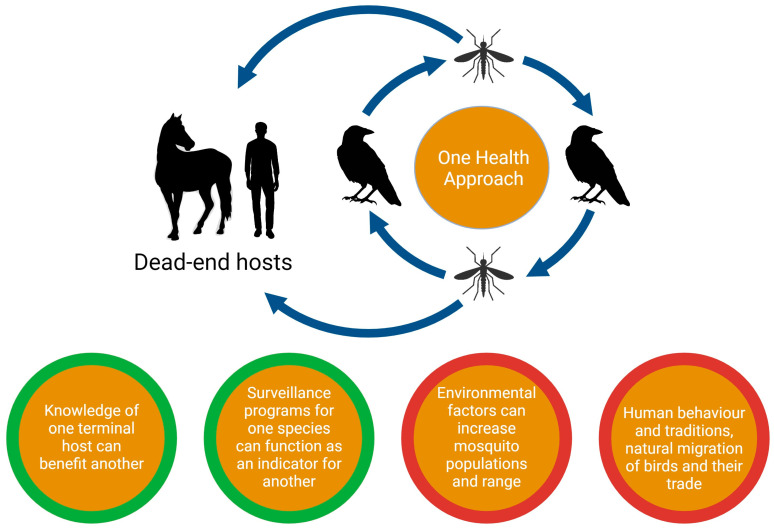
West Nile virus (WNV) lifecycle, and possible positive (green outline) and negative (red outline) implications on the whole system, illustrating the potential benefits of using a One Health Approach.

The family *Flaviviridae* consists of four genera, *Orthoflavivirus*, *Hepacivirus*, *Pestivirus*, and *Pegivirus* [1]. These genera include numerous human and veterinary pathogens of clinical relevance. For example, hepatitis C virus (HCV) belongs to the genus *Hepacivirus*, while West Nile virus (WNV), yellow fever virus (YFV), and dengue virus (DENV) belong to the genus *Orthoflavivirus* [2]. The genus *Orthoflavivirus* includes 53 species that can be further divided into mosquito-borne and tick-borne viruses. Both mosquito-borne and tick-borne viruses are substantially involved in many human and veterinary diseases [3,4]. Mosquito-borne viruses can be categorized into encephalitic or non-encephalitic clades such as WNV and dengue, respectively [5]. Pathogens from the genus *Orthoflavivirus* are globally distributed affecting all the continents except Antarctica [6]. Migrating birds are the amplifying host and the source of viral spread over great distances, whereas humans and equines are dead-end hosts (Figure 1) [6,7]. Interestingly, neuroinvasiveness is the main characteristic of a number of *Orthoflavivirus* infections, including WNV infection [6,8]. Due to the global health impact of disease caused by WNV in both humans and veterinary species, WNV infection is listed as notifiable by multiple global and local health organizations. WNV infections detected by national surveillance systems should be reported to the World Health Organization (WHO) if there is a serious public health impact or if an outbreak is unusual or unexpected, when combined with a significant risk of international spread [9]. West Nile fever (WNF) is a listed disease for multiple veterinary species within the World Organization for Animal Health (WOAH) Terrestrial Animal Health Code, which means that infection, even in the absence of clinical disease, must be reported to the WOAH by all 183 member countries [10]. Schwarz and Long [11] provided a comprehensive review which compares WNV disease in humans and horses, and describes the implications for diagnostics and syndromic surveillance. The current review additionally describes the extensive epidemiology and prevalence in humans and equines, an immunological comparison between humans and equines, One Health approaches, and proposes the use of WNV infection in equines as a model for human WNV infection.

## 2. Epidemiology and Prevalence of WNV in Equines and Humans

WNV was first identified in East Africa from the blood of a Ugandan woman in 1937 [12] and remained restricted to Africa and Asia until the end of the 20th century [13,14]. Since then, WNV infections in equines and humans have been reported worldwide; so far, all continents, except Antarctica, have experienced outbreaks of WNV [15,16,17]. It is expected that WNV infection will become even more widespread due to global climate change. Changes in weather conditions such as temperature, precipitation, humidity, and wind can have a wide variety of effects from changing the habitat range of WNV susceptible mosquito species to a change in viral transmission efficiency and viral replication rates [18]. WNV can be divided into nine phylogenetic lineages; however, lineages 1 and 2 are considered the most important lineages because of their widespread occurrence, pathogenicity, and their involvement in multiple global outbreaks in humans and equines [15]. Lineage 1 can be further divided into clade 1a and clade 1b; clade 1b has been isolated in Australia. Lineage 1 clade 1a can be further divided into clusters 1–7 which, historically, have been found in various countries worldwide [19,20].

Equine and human WNV infection has been reported in various countries within North, Central, and South America [15]. According to the United States Department of Agriculture (USDA), more than 28,000 equine WNV cases have been confirmed since lineage 1a first disseminated to the United States of America (USA) in 1999 (Table 1) [21,22]. During the initial outbreak in 1999, 62 people tested positive for WNV, of which 7 died [23]. After the dissemination of WNV in North America, the same WNV strain was later reported in Central and South America. This strain appeared to have a close genetic relationship with WNV strains found in Israel suggesting its Middle Eastern origin [24,25,26]. In the USA, by 2000, WNV had expanded to twelve states as well as to the District of Columbia [26]. It is estimated that 7 million people across the USA were infected with WNV between 1999 and 2016, of which 2017 people died (Table 1) [27]. Based on the data collected between 1999 and 2022, the yearly incidence rate of people contracting WNV neuroinvasive disease is 1.07 per 100,000 people [28]. For equines, the largest outbreak of 15,257 confirmed cases, involving 43 states, was reported in 2002 and is estimated to have cost the USA’s economy USD 1.5 million; most cases were confirmed in central USA [29]. During this outbreak, a mortality rate of 22% was reported based on 569 confirmed cases within North Dakota [30]. The availability of the WNV vaccine for equines in late 2002 resulted in no further increase in equine cases and a decrease in cases was even reported in 2003 [31]. However, there was a large increase in human cases in 2003 due to the unavailability of an effective vaccine (Table 1). Furthermore, in the years after the introduction of the equine WNV vaccine, the number of equine cases dropped even further, and the cases that are reported tend to be in unvaccinated and under-vaccinated equines according to the USDA [32]. The success of vaccination is also demonstrated in Canada, where 852 cases of equine WNV infections were reported to the Canadian Food Inspection Agency (CFIA) between 2003 and 2019. The majority (96%) of these reported Canadian cases were unvaccinated equines with a mortality rate of 31.9% [33]. Sporadically, an unexpected increase in equine cases can be observed, which seems to be correlated with an increase in human cases. For example, in 2012, the number of reported equine cases in Texas was 121 compared to only 6 cases the year before. Similarly, in 2012, the number of human cases reported in Texas was 1,868, compared to only 27 cases in the year before [34,35]. However, no information is available regarding the vaccine status of the affected equines.

Throughout Central and South America, lineage 1a WNV infection-related mortality and morbidity in equines, avian, and humans have been reported. However, WNV is much less prevalent in comparison to North America, despite the similar strains that circulated in those areas [22,38]. Major outbreaks have so far not been reported despite suitable circumstances that are able to support the establishment and preservation of WNV transmission. Various hypotheses have been proposed for this phenomenon including under-reporting, misdiagnosing of viral diseases with a similar presentation, cross-protection provided by infection of other *Orthoflavivirus* species, decreased virulence of WNV due to genomic mutations, and competition between WNV and Saint Louis encephalitis virus [17,39].

In Europe, the first lineage 1a WNV outbreak involving humans and equines was reported in 1962, lasting up to 1965 in the Camargue wetlands of France, a great breeding location for mosquitos. The first sign of an outbreak in 1962 was the detection of neuroinvasive disease in wild and domestic horses, leading to a case mortality rate of 25–30% [40,41]. Based on serological surveillance, it is expected that WNV remained present after 1965 at very low levels in this region until the next case in 2000 [42]. Hereafter, the first major outbreak involving neuroinvasive disease in humans was reported in Romania in 1996. A total of 393 cases of WNV infection were confirmed through serological assays. Of these, 352 people experienced neuroinvasive disease and 17 people died [43]. Of these confirmed cases, 286 (73%) were located in the city of Bucharest. Based on serological surveys, it is expected that during this outbreak more than 90,000 residents were infected; therefore, less than 1% experienced neuroinvasive disease [44]. During this outbreak, no increase in cases of equine encephalitis was reported to the Ministry of Agriculture of Romania [43]. Another major outbreak occurred in southwest Russia in 1999, resulting in 800 human cases with 40 deaths [45]. In between major outbreaks, a limited number of cases were reported yearly in various countries in southern and eastern Europe. In 2004, lineage 2 WNV was first reported in Europe, when a goshawk with lethal encephalitis was found in Hungary. This isolate was shown to have the closest phylogenetic relationship with lineage 2, isolated in central Africa [46]. Once thought to be of low pathogenicity due to high seroprevalence rates in equines without disease presentation in South Africa [47], lineage 2 WNV has now been reported to cause neuroinvasive disease in humans, equines, and livestock species [48]. At the moment, lineage 2 WNV is the main lineage causing seasonal outbreaks in Europe, with only occasional reports of lineage 1 virus isolation [42]. A major increase in cases was seen in 2018. A total of 1548 locally acquired human cases were reported, of which 166 people died. The majority of these cases were reported in Greece (39%), Italy (20%), and Romania (18%) [49]. Since 2016, equine cases are also monitored by European Centre for Disease Prevention and Control (ECDC), they reported 285 equine cases during the major human outbreak in 2018 [50]. Only a few years later, in 2022, another major outbreak was reported by the ECDC, with 1340 human cases leading to 104 deaths. The majority of cases were again reported in Greece, Italy, and Romania. Together, 9 European countries reported 101 equine cases, the majority of cases were reported in Italy (47%). Unlike human cases, the second biggest portion was reported in Germany (16%) which might be related to a difference in equine surveillance strategies and equine populations of various countries [51]. Overall, in Europe, WNV infections in humans and equines have been mostly reported in countries in Southern Europe near the Mediterranean Basin, such as Bulgaria, Croatia, Greece, Italy, Macedonia, Morocco, Portugal, Romania, and Spain [52,53]. However, sporadic cases and outbreaks in humans and equines have, over time, been reported more frequently in countries further north, as far up as Germany and The Netherlands [54,55].

WNV infection in humans and equines in Africa has been reported; however, due to a lack of epidemiological data for a substantial number of countries, the historical and current spread is not entirely understood. Mencattelli et al. [15] have recently provided a comprehensive review describing the past and current epidemiological status of WNV infection in humans, equines, and other animals within Africa. Briefly, WNV has been reported in at least 28 African countries since it was first identified. Predominantly, lineage 1, 2, or a mixture of these two, have been reported in various countries throughout Africa. Furthermore, in 1992, lineage 8 was reported in Senegalese mosquitos, but so far, no human or veterinary cases have been identified. This is likely due to the low virulence of lineage 8. For humans, the first case of WNV infection was described in 1937 and many cases have been described ever since. Reported cases are spread over the entire continent with major outbreaks being described from the 1950s onward. In more than 20 African countries, no serological assays in humans have been performed; therefore, the actual burden on the human population is likely severely underestimated. Records of equine WNV infection are spread over the entire continent as well, and go back to 1963, to a study in Egypt. In 1996, an outbreak in Morocco involving 42 deaths was described. Since then, many records have been published describing deaths among equines [15].

Equine and human WNV infections have been well reported in the Middle East, especially in countries within the Mediterranean Basin, but are less commonly reported in the other parts of Asia. The first recognized human WNV epidemic occurred in 1951 in Israel. Hereafter, an outbreak in an Israeli nursing home in 1957 with a high incidence (33%) of neuroinvasive disease was reported. Subsequently, major outbreaks were not reported for a number of decades until the next epidemic in 2000 [56]. Since then, WNV has spread further east. Based on serological assays it is expected that WNV infection was prevalent in Pakistan in the 1970’s. However, it was not until the epidemic of 2015–2016 that WNV neuroinvasive disease was confirmed in humans [57,58]. Further, Sri Lanka confirmed WNV infection in three patients who were admitted to hospitals while presenting with encephalitis or meningoencephalitis in 2013 [59]. Based on serological assays, it is expected that WNV has also been prevalent in India since at least 1952. Since then, multiple outbreaks involving encephalitis and acute flaccid paralysis have been reported, which have been attributed to WNV lineage 1 and 5 [20,56]. Interestingly, in western countries WNV infection fatality is related to increasing age, with higher fatality rates among elderly patients; however, in India, a relatively high number of pediatric fatalities related to acute encephalitis syndrome have been reported which have been attributed to lineage 5. In total, there is serological evidence of WNV infection in humans in at least six Middle Eastern countries, including Israel, Jordan, Yemen, Iran, Iraq, Turkey, and five Asian countries located further east, including Pakistan, Afghanistan, India, Sri Lanka, and China [56,60]. Similar to humans, seroprevalence in unvaccinated equines has been reported in the Middle East. For Israel, a seroprevalence of 84% was reported in 2018 [61], while for Pakistan, a seroprevalence of 65% was identified [62]. In addition, Jordan (25%) and Saudi Arabia (17–56%) have reported similar high seroprevalence rates indicating substantial circulation of WNV in equines in these parts of Asia [63,64]. In Israel, WNV infection of equines is considered endemic in geographical regions that align with major bird migration routes, and cyclic epidemics are observed in the rest of the country [65]. Less is known about the seroprevalence of WNV in equines in more eastern Asian countries. Seroprevalence has been reported in India (3.2–3.4%); however, serological research of equines in Shanghai, China failed to detect any seroprevalence of WNV (0%) [66,67].

In Australia, Kunjin virus, which belongs to lineage 1b WNV, was first identified in 1960 in *Culex annulirostris* mosquitoes originating from North Queensland [68]. In 1964, serological assays showed evidence of infection in humans, various avian species, horses, cattle, and kangaroos [69]. Kunjin virus infection is generally mild or asymptomatic, the first human case involving encephalitis was confirmed in 1974, after which only sporadic cases have been confirmed without major outbreaks in humans [70]. Although on average only four cases are reported annually, the annual infection rate is estimated at 0.15% in Queensland, based on blood donor serological screening. This leads to an estimated annual infection of 14,760 people [71]. Even though human outbreaks have so far not been reported, interestingly, a major outbreak involving encephalitis in equines was reported in 2011. During this outbreak over 1,000 horses were affected, with a mortality rate of 10–15% [72]. Clinical signs were similar to those reported for the horses infected with WNV in the USA between 2000 and 2001, including muscle paralysis and tremors, weakness of the limbs, ataxia, incoordination, and changes in temperament. The outbreak was most likely not related to an imported WNV strain since the isolated strain was genetically closely related to indigenous Kunjin virus strains, not to exotic strains [73]. So far, New Zealand remains WNV free despite having susceptible *Culex* mosquito species [74,75].

Globally, the number of equine cases is likely underreported due to a lack of routine testing programs. Since only 10–39% of infected unvaccinated equines show clinical signs, the majority of unvaccinated equines that do not experience clinical signs will likely not be reported [76]. Therefore, the prevalence and routine surveillance of WNV in equines needs more attention. Moreover, WNV is spreading to geographical areas that previously remained WNV free and is becoming endemic to new areas where only sporadic cases were noticed previously. In conclusion, WNV has been reported on all continents, except Antarctica, and affects both equines and humans. WNV lineage 1 and 2 are found in humans as well as in equines. Either lineage 1 or 2, or a mixture of the two, can become dominant strains, depending on the geographical location.

## 3. Transmission of WNV

WNV is transmitted from infected mosquitos to birds through probing during blood feeding. In birds, WNV can reach viremia levels that are transiently high enough for transmission when another susceptible mosquito feeds on the same bird [77]. Birds are considered the natural reservoir or primary amplifying host of WNV, approximately 100 bird species have been described to be involved in WNV infection and transmission [77,78]. Humans and equines are dead-end hosts that can be infected with WNV originating from mosquito bites (Figure 1). However, viremia does not reach high enough levels for transmission to occur from these hosts to another mosquito [79]. In humans, titers can reach 0.06–0.50 plaque-forming unit (PFU)/mL with a median of 0.1 PFU/mL based on viremic blood donors [80]. However, these viremic blood donors might not have been at their peak viremia levels at the moment of blood donation; therefore, the actual peak levels of viremia might be higher. A study investigating 245 viremic human blood donors for a prolonged period of time showed a median time of 13.2 days until WNV ribonucleic acid (RNA) negativity was measured; however, for four donors, the WNV RNA persisted over 40 days [81]. During an equine infection experiment, viremia titers ranged between 10^1.0^ and 10^3.0^ PFU/mL, which were measured for 6 days post-infection. In this experiment, 10^1.0^ PFU/mL was the lowest measurable titer. Therefore, in reality, titers might have been lower but undetectable with the methods used [82].

In the USA, mosquitos of the genus *Culex*, especially *Culex pipiens, Culex tarsalis*, and *Culex quinquefasciatus*, are considered the primary vectors in disease transmission; however, WNV has been identified in a total of 65 species of mosquitoes from diverse environments [83]. It remains unclear how many of the mosquito species that can be infected can also transmit WNV and if so, to what extent. For example, Goddard et al. [84] researched ten *Culex* species that are present in California and found that all of the mosquito species were able to be infected and were able to transmit disease to some extent. Transmission rates depended on the mosquito species, the original infection dose of the mosquito, and the number of days after mosquito infection at which a transmission attempt was performed [84]. The mosquitoes inject their saliva into the skin to liquefy the tissue for efficient probing. The immunomodulatory effect of mosquito salivary proteins reduces the functionality of host immune cells such as T helper 1 and 2 cells (Th1 and Th2) and reduces the production of interferon beta (IFN-β) and nitric oxide (NO) by macrophages [83,84,85,86,87]. Thus, alteration in immune functions by mosquito salivary proteins enhances the infectivity of WNV, and many reports describe that WNV persists at the injection site in the skin for up to two weeks post-infection [83,88]. Although there are many reports on the involvement of salivary proteins in WNV transmission and enhanced infectivity of the virus, additional research is required to explore the salivary factors enhancing WNV infectivity during the early phase of infection. Non-vector-related transmission mechanisms of WNV through blood transfusions, organ donation, and breastfeeding have been described, but are not considered the principle routes of viral transmission [89,90].

## 4. Susceptibility of Equines and Humans to WNV Infection and Disease

Susceptibility to WNV infection and disease development can be determined through a number of risk factors. A number of studies report a relation between WNV susceptibility and the breed of horses. However, this factor is closely related to the various ways of managing the living conditions of these horses which is likely a true risk factor [91,92,93]. A study performed in Germany studying risk factors in equines (by their definition horses, donkeys, and mules) used equines of at least 12 months of age that were unvaccinated. Their study population included 940 equines of which 54 (5.8%) were shown to be seropositive for WNV. Equines within holdings with higher vaccination densities had a reduced chance for seropositivity (odds ratio (OR) = 0.97). This possibly indicates some kind of herd immunity provided by vaccination. Risk factors that increased chances of seropositivity included the following: being in counties where previous equine WNV infections had been reported (OR = 3.91), housing conditions such as permanent outdoor housing (OR = 2.63), having a shelter in the turn-out (OR = 3.02), and using fly sheets (OR = 7.22) [92]. Regarding a genetic basis for WNV susceptibility, genetic variation in the regulatory region of an IFN-induced gene 2′-5′-oligoadenylate synthetase 1 (OAS1) has been related to increased susceptibility to encephalitis [94]. The protein OAS1 is involved in the molecular pathway of RNase L activation which degrades, among others, viral RNA [95]. Furthermore, a study looking into the relations between genetic variation and WNV-induced encephalitis in Camargue horses found a correlation between compound two genotypes of single nucleotide polymorphisms (SNPs) and immune-related candidate genes such as mitochondrial antiviral-signaling protein (MAVS), natural cytotoxicity triggering receptor 2 (NCR2), and interleukin 10 (IL-10). This study also identified two microsatellites (HMS082 and CZM013) which were related to WNV-induced encephalitis [96].

Host risk factors for humans include their age, sex, nutrition, hormonal, and immune status which affects the outcome of infection. Moreover, a person’s living environment is considered to be a risk factor. People in humid regions are more likely to be seropositive in comparison to people living in colder and highland regions. The risk of WNV-induced encephalitis in people increases with age and other underlying medical conditions such as diabetes [97]. The development of WNV neuroinvasive disease is related to many environmental and host risk factors. Various human genes have been identified as being associated with the increased or reduced risk of neuroinvasive disease development. The genetic aspects of neuroinvasive disease development are complicated and multifactorial. For example, variation in alleles within the viral immunity-associated human leukocyte antigen-A (HLA-A) and HLA-DQB1 loci can both enhance and reduce the risk of neuroinvasive disease. Furthermore, multiple SNPs within the HECT And RLD Domain Containing E3 Ubiquitin Protein Ligase 5 (HERC5) gene have been related to neuroinvasive disease susceptibility [98]. Although the exact antiviral mechanism of HERC5 expression has not been established for WNV, it has been shown to have a wide variety of antiviral activities. For example, by restricting the early-stage assembly of HIV-1, by attenuating influenza A through non-structural protein 1 (NS1) modifications, and by inhibiting human papillomaviruses [99,100,101]. In addition, variation within an intergenic region between cluster of differentiation 83 (CD83) and jumonji and an AT-rich interaction domain containing differentiation 2 (JARID2), and more rarely, SNPs within the loci of TFCP2L1 and CACNA1H were associated with the development of neuroinvasive disease. The potential antiviral or viral-stimulating properties within variations of TFCP2L1 and CACNA1H has not been established [98]. Similar to equines, variation within the multiple OAS-related genes has been shown to alter WNV resistance and susceptibility, although their molecular mechanisms have not been established [98,102,103]. Variation within a single SNP of the OAS1 gene has been strongly associated (OR = 9.79) with increased susceptibility to encephalitis and paralysis induced by WNV infection. Finally, symptomatic WNV infection has been associated with multiple SNPs within the interferon regulatory factor 3 (IRF3; OR = 0.54) and MX dynamin like guanosine triphosphate(GTP)ase 1 (MX1; OR = 0.19) genes. IRF3 plays a role in the upregulation of type I IFN genes, while MX1 encodes a GTPase with antiviral properties. Nevertheless, the exact mechanism of how these genes alter WNV infection susceptibility is not clear, which warrants further investigation [104].

## 5. Comparative Clinical Manifestation and Pathogenesis of WNV Infection in Humans and Equines

Based on the current available information, equines and humans share a similar clinical manifestation and pathogenesis upon WNV infection. However, the clinical manifestation and pathogenesis of equine cases with asymptomatic or mild infection is not as clearly described as the corresponding human infections. Moreover, it is important to acknowledge that equines cannot express their feelings and symptoms like humans can. For example, humans can express having a headache, while equines cannot. On the other hand, experimental infections using humans is not an option; therefore, human information can be more difficult to interpret due to many varying parameters of case studies such as infection strain and dosage, patient age, and so on. A substantial part of information known to date regarding WNV infection is derived from experimental infection of equines and severe equine cases involving neuroinvasive disease. In 1963, the first experimental equine WNV infection article was published [105]. Since then, more equine infection experiments using various methods of infection such as intradermal, subcutaneous, intrathecal, and mosquito-feeding have been performed. Due to the similarities between human and equine viral kinetics, these studies have aided the understanding of WNV infection dynamics [82,106,107,108,109].

In general, the clinical manifestation of WNV infection in humans and equines can be divided into the three following categories: asymptomatic infection; symptomatic infection without neuroinvasive disease that is generally characterized by fever, which is also known as ‘West Nile fever’; and symptomatic infection with neuroinvasive disease which includes aseptic meningitis, encephalitis, and acute poliomyelitis-like syndrome [110]. In the literature, varying incubation periods between 3 and 14 days have been described for humans, but the overall median is 2.6 days [111]. However, for both humans and equines, the majority of infections are asymptomatic. It is expected that, based on large scale blood donor screening, 74–79% of human cases are asymptomatic. Therefore, an estimated 21–26% of people infected with WNV become symptomatic, of which almost half will seek medical attention. However, only 5% of the total number of infected people are estimated to receive a WNV infection diagnosis [112,113]. Clinical signs of symptomatic infection without neuroinvasive disease in order of occurrence frequency include, but are not limited to, fatigue (96%), fever (81%), headache (71%), muscle pain (62%), muscle weakness (61%), rash (57%), neck pain or stiffness (55%), difficulty concentrating (52%), joint pain or aches (37%), vomiting (28%), diarrhea (27%), and sensitivity to light (21%). The most characteristic symptom of symptomatic infection without neuroinvasive disease is fever, which lasts for 5 days on average. Residual symptoms most commonly include fatigue and muscle weakness and are reported by 63% of patients 30 days after the onset of disease [114]. Less than 1% of the total number of humans infected with WNV are estimated to develop neuroinvasive disease [115]. In addition to the clinical signs associated with symptomatic infection without neuroinvasive disease, symptomatic infection with neuroinvasive disease is associated with inflammation of the tissues in the spinal cord and brain which can cause various manifestations such as meningitis, encephalitis, and acute poliomyelitis-like flaccid paralysis [116,117]. The most common neurological clinical signs observed in patients upon initial assessment are tremor (35%), cognitive impairment (45%), cranial neuropathy including facial weakness (16%), and coma (25%). Various forms of weakness occur in 49% of patients with neuroinvasive disease. These forms of weakness include generalized weakness (74%), asymmetric limb weakness (15%), weakness in upper extremities (7%) and paraparesis (4%) [117]. Ocular manifestations, including blindness, chorioretinitis, anterior uveitis, vitritis, and retinal hemorrhage have been well-documented in humans [118]. In addition, some rarer clinical manifestations which have less established causal relationships to WNV infection are rhabdomyolysis, hepatitis, pancreatitis, myocarditis, cardiac arrhythmias, and renal failure [110,119]. Among people that develop neuroinvasive disease, the reported mortality rate is between 10% and 30% depending on age and immune status, which represents less than 0.1% of all infected people [110,117]. Approximately 60% of people that recover from neuroinvasive disease experience long-lasting effects 90 days after their initial evaluation such as tremor (23%), various forms of weakness (48%), cognitive impairment (19%), cranial neuropathy (8%), and coma (2%) [117].

The clinical manifestation of equines is similar to human cases, it is estimated that approximately 10–39% of infected equines will develop clinical disease. Therefore, the majority of cases are asymptomatic [76]. However, neuroinvasive disease is more common in equines in comparison to humans, with 8–10% of cases developing neurological signs [82]. For equines, symptomatic infection involving neuroinvasive disease is well documented and studied. However, cases with symptomatic infection without neuroinvasive disease are not well documented. This is likely due the milder clinical manifestation that generally does not require immediate veterinary care. Based on experimental infection, it is expected that the incubation period between infection and the first neurological signs is 7–9 days [82,108]. The most common clinical signs observed for confirmed cases are ataxia (75%), muscle weakness and paralysis (55%), fever (48%), anorexia and lethargy (28%), abnormal behavior including hyperesthesia, teeth grinding, hydrophobia, anxiousness, and circle walking (20%), muscular tremors and twitching including generalized tremors, localized tremors and twitching of the lips and eyelids, and jaw muscle spasms (15%), and cranial nerve deficits including blindness, difficulty swallowing, and facial paralysis (14%) [120]. Ophthalmic changes have been reported in equines as well, although blindness is considered one of the rarer clinical signs, incidence rates as high as 37.5% of cases have been reported [121]. More commonly, incidence rates of 14% and lower are reported [120]. Further, additional clinical signs that can be observed are as follows: enterocolitis; colic; rectal prolapse; lameness, which can present itself in various ways including a laminitic stance; cervical and thoracic pain; anemia; inflammation of the tongue; icterus, indicating hepatic involvement; and urinary dysfunction, indicating renal involvement [120,122,123,124,125]. In conclusion, equines experience a similar clinical manifestation as humans, although the classification systems of equine and human clinical manifestations differ due to multiple limitations regarding the expression and measurability of symptoms and clinical signs in equines and humans. Equines usually recover naturally, but may require extensive supportive care, including the use of slings to hold them upright at the peak of their ataxia and muscle weakness [126]. The mortality rates of equines that develop neuroinvasive disease vary in different studies with mortality rates as high as 71% reported [127]. However, mortality rates of 22–36% are more commonly reported [29,82,128]. Mortality rates are higher for older and immunocompromised equines [22,30]. In addition, one study reported a higher mortality rate in foals under 12 months of age (66.7%) [129]. Like humans, equines that do recover from neuroinvasive disease can experience long-lasting side effects as illustrated by the 10–20% of cases exhibiting residual neurological deficits [128]. Porter et al. [126] reported that most of the horses with encephalomyelitis had residual deficits one month post-discharge from the veterinary hospital, which resolved for 90% of the horses by 12 months after discharge.

Clinical pathology plays a crucial role in understanding and managing WNV infections in both human and equine patients. After infection, WNV can spread in both retrograde and anterograde directions of neurons through axonal transport. Axonal transport facilitates dissemination into the central nervous system (CNS) and facilitates transneuronal spread through axonal release of viral particles [130]. In humans, the CNS most often exhibits significant impacts on regions related to movement, such as the extrapyramidal system. Although all parts of the brain can be affected by neuroinvasive disease, most commonly the brainstem (specifically the medulla and pons), deep gray matter nuclei (comprising the basal ganglia and substantia nigra of the thalamus), and the gray matter encompassing the cerebellum are affected [131,132,133,134,135]. The most widely recognized and extreme lesions are found in the thalamus, basal ganglia, pons, and medulla oblongata [136]. In the spinal cord, the anterior horns (ventral horns) and posterior spinal nerve roots are often involved and associated with lower motor neuron loss resulting in muscle weakness [131,132,137,138]. Clinically observed muscle weakness correlates histologically with neurogenic atrophy of skeletal muscle [138]. Hematological test results show that anemia (41.1%), as illustrated by a mild decrease in hemoglobin levels, is common in patients experiencing West Nile fever. In addition, both leukocytosis (35.9%) and leukopenia (8.6%) can be observed, as well as thrombocytopenia (14.9%) and hyponatremia (33–50%) [139,140,141,142]. Laboratory analysis of cerebral spinal fluid (CSF) shows severe pleocytosis for patients with meningitis (97%), encephalitis (95%), and especially acute flaccid paralysis [116,143]. Protein levels are increased in patients experiencing acute flaccid paralysis, and in contrast, they are decreased in patients experiencing meningitis, while remaining unaffected in patients with encephalitis. In addition, the number of lymphocytes is increased in the CSF of patients experiencing acute flaccid paralysis [116,126,136,144,145]. Neutrophilic pleocytosis, defined as a predominance of neutrophils greater than 50%, can be observed in patients with meningitis (45.4%) and encephalitis (36.9) [146,147]. The pathology of long-lasting WNV infection of the brain mimics the pathology found in people with neurodegenerative diseases such as Alzheimer’s’ disease and Parkinson’s disease. This may help to explain why long-lasting effects of infection result in cognitive impairments. Long-lasting inflammation of the brain resulting from WNV infection leads to damage and dysfunction of neurons. Ultimately, this inflammation leads to apoptosis of neurons, accumulation of misfolded proteins, a decrease in neurogenesis, and activation of microglial cells leading to the formation of microglial nodules and phagocytosis of presynaptic terminals of neurons [148].

The clinical pathology of equines has been researched through tissues of naturally infected equines submitted to hospitals, and more commonly through studying tissues of experimentally infected equines [107,108,121,125]. The pathology of equine WNV infection is very similar to human WNV infection and a comparison of the pathology of these two species has been well reviewed by Schwarz and Long [11]. Moderate to severe nonsuppurative polio encephalomyelitis in the brain and spinal cord is the histopathological hallmark of WNV neuroinvasive disease in equines and humans [149,150]. For equines, the spinal cord and the gray matter of the midbrain and hindbrain are commonly affected, and the cerebral cortex seems to be least affected [126]. Visible lesions in the CNS that can be observed are mild to moderate meningeal hyperemia, subdural exudates with fibrin tags, and focal areas of hemorrhage within the brainstem and spinal cord [151,152,153]. Furthermore, cell death through pyroptosis (fiery cells) can be found most commonly in the thalamus, basal ganglia, midbrain, and hindbrain [129,151,154]. Gliosis and the formation of glial nodules in combination with neuronal degeneration and limited necrosis has been observed. Spinal cord lesions are limited to the gray matter of the ventral and lateral horns in equines and the foremost horns in humans. In certain equine cases, lesions can be exclusively limited to the spinal tissues [126,151]. Affected spinal cords histologically resemble those of humans with polio-encephalomyelitis, containing lymphocytes, small numbers of macrophages, and neutrophils in vessels with leukocyte cuffing, glial nodules, and occasional neuronophagia [126,151]. Similar to that observed in humans, WNV in equines shows a predilection for cranial nerve motor neurons of the mid- and hindbrain, leading to clinical signs such as difficulty with swallowing, drooling, and unilateral facial paralysis [11,152,155,156]. Ataxia, weakness, and paralysis can be attributed to motor neurons with infection in the gray matter of the spinal cord. The most common hematological observation that can be made is a decrease in peripheral lymphocyte count. In addition, although less common, an increase in neutrophil counts can be observed as well [11]. It is unclear what the roles of neutrophils are in this context as they can play both protective and detrimental roles during infection [157]. CSF can be hard to obtain in severely affected equines due to neurological abnormalities; therefore, only a limited number of studies discuss CSF laboratory analysis [121]. The pathology observed regarding the CSF of equine patients is similar to the clinical pathology observed for the CSF of human patients. Severely affected equine patients display mononuclear pleocytosis that is lymphocytic, and sometimes neutrophilic pleocytosis is displayed [126,144]. Laboratory analysis of CSF can provide highly variable results. In eight naturally infected horses suffering from encephalomyelitis, differential leukocyte counts varied from normal counts, counts indicating suppurative or nonsuppurative inflammation, and lymphocytic pleocytosis. There was a mild to moderate increase in the total protein concentration in three horses [125]. One study reported a low leukocyte count in the CSF of a horse presenting with encephalomyelitis caused by natural infection, which is relatively normal. However, a high total protein concentration and xanthochromia were also observed in the CSF [121]. Thus, overall, both the clinical manifestation and clinical pathology of human and equine patients that have been observed so far is very similar.

## 6. Comparative Immune Responses to WNV in Equines and Humans

Upon infection, both innate and adaptive immune responses can be attributed to the rapid clearance of WNV which has, for humans, been well reviewed by Samuel and Diamond [158]. One limitation of this review is that the description of the immunological data relies heavily on experiments performed on mice, who are not natural hosts of WNV. Innate immune cells that play an important role in WNV infection are macrophages, dendritic cells, and natural killer (NK) cells. Macrophages play a role through phagocytosis, antigen presentation, production of NO intermediates, and secretion of chemo- and cytokines [158]. Based on murine models, it was determined that during infection dendritic cells express MAVS which leads to cleavage of viral RNA [159]. Dendritic cells also function as important antigen-presenting cells. However, WNV infection of human-derived dendritic cells leads to a decrease in the molecules needed for antigen presentation and a decrease in the expression of molecules involved in T cell co-stimulation [160]. NK cells kill virus-infected cells that fail to express major histocompatibility complex class I (MHC-I). However, killing by NK cells is only transient during WNV infection, indicating an evasion mechanism, possibly through virus-induced upregulation of MHC-I [158]. During WNV infection, the innate immune system uses all three complement pathways (classical, alternative, and lectin), deficiencies in these pathways impair T cell responses and the production of specific antibodies [158]. Additionally, in humans, IFNs play an important role during viral infection. Most cell types can produce IFN type I (IFN-α and IFN-β) and upregulation hereof leads to the induction of an antiviral state and stimulates maturation of dendritic cells which provide a link between the innate and adaptive immune system [158]. Type II IFN (IFN-γ) is primarily produced by NK cells, Th1 CD4+ T cells, γδ-T cells, and CD8+ T cells upon viral infection and plays an important role in the combating of viral disease in both the periphery and the CNS. Upregulation of IFN-γ leads to upregulation of MHC-I and -II which enhance the immune response. Furthermore, IFN-γ can activate macrophages in the periphery and microglial cells in the CNS, and can induce antiviral pathways thereby limiting infection [158,161]. IFN-γ specifically plays a role in WNV infection by limiting viral dissemination in the CNS during the early stage of infection. This limiting effect is predominantly carried out by γδ-T cells and not CD8+ T cells [158]. Various receptors within the innate immune system play antiviral roles. Based on studies using mice and mice cell lines it was determined that both retinoic acid-inducible gene-I (RIG-I) and melanoma differentiation-associated protein 5 (MDA5) can sense WNV RNA leading to the activation of MAVS. MAVS activation in turn leads to the activation of IRF-3, and the induction of IFNs and interferon-stimulated genes (ISGs) [162]. These genes include viperin, interferon-induced protein with tetratricopeptide repeats (IFIT), and other genes that suppress viral replication and infection. However, WNV can overcome the host’s restriction by IFIT proteins through 2’O methylation of its RNA, as demonstrated in mice [163]. Furthermore, based on data derived from studies using genetically deficient human fibroblasts and mice, the WNV protein NS5 functions as an IFN-α and IFN-β antagonist which can prevent the release of IFN-α receptor 1 (IFNAR1) from host cells leading to inhibition of the IFN response [164,165]. In addition, the innate immune response utilizes the OAS and RNase L pathways to restrict WNV infection. In humans, a single substitution of C to T in the OAS1b gene is critical for the development of encephalitis and paralysis in WNV infection [166]. WNVs double-stranded RNA is detected by OAS proteins which undergo a conformational change to synthesize 2′-5′-oligoadenylates that ultimately bind to inactive RNase L, thereby activating it and leading to the cleavage of viral RNA [145].

In humans, regarding the adaptive immune system, T cells play an essential role in the clearance of WNV infection. This is shown in people with hematological and T cell abnormalities who are at increased risk of developing neuroinvasive disease. Cytotoxic T cells (CD8+) are able to proliferate and kill infected cells upon recognizing the WNV antigen presented on MHC I. The presence of CD8+ T cells in the CNS has shown to be essential for viral clearance. In addition, for a sufficient specific humoral response, involvement of CD4+ T cells is required. Deficiencies in this pathway, like deficient MHC II, leads to an increased mortality and increased viral persistence in the CNS [158]. Immunoglobulin M (IgM) is the first antibody isotype produced upon primary infection and is of great importance during the early humoral immune response. Research using soluble IgM double knockout mice showed increased viremia levels, increased WNV dissemination into the CNS, and increased mortality compared to wild-type mice. Mortality of wild-type mice could be predicted based on WNV-specific IgM titers at 4 days post-infection; lower WNV-specific IgM titers lead to higher mortality rates [167]. Often the presence of IgM is used as an indicator of acute WNV infection in human patients. However, IgM expression in CSF has been observed for 199 days post-acute infection and might not, therefore, always provide an accurate indication of disease progression [168]. Although it is known that IgG can play a protective role during WNV infection, it is less clear what the exact mechanism of this is during primary infection. One challenge is that it takes 6–8 days for IgG to be produced which is after the clearance of the virus in the periphery and after the dissemination of the virus into the CNS [158]. It has been shown that following WNV infection, IgG1 is the predominant IgG subclass produced in humans. In comparison to IgG2 and IgG3, IgG1 is the subclass that almost solely contributes to in vitro virus-neutralizing capacity measured in Vero cells, In vivo, it also shows superior effector functions in mice infected with WNV that have been treated with human-derived IgG [169]. Humanized and human-derived antibodies can have a protective role through their virus neutralizing capabilities in vitro. It has been shown that neutralizing antibodies are capable of blocking the transneuronal spread of WNV by neutralizing viral particles released by axons [130]. Most murine-neutralizing antibodies bind to domain III of the envelope (E) protein; however, neutralizing antibodies with high affinity to domain I and II of the E protein have also been observed [170]. Interestingly, the neutralizing capabilities of human-derived antibodies seem to be dependent on both time and temperature. Given the right circumstances, even epitopes that are generally thought to be inaccessible can be utilized [171]. Passive immunity can be used to protect the host from WNV infection. Treating antibody and B cell activating factor receptor (BCAFR) deficient mice with immune sera antibodies from wild-type mice was successful in the development of sustained protective immunity from WNV infection [144].

As seen in humans, in equines both the innate and adaptive immune system are activated which can lead to the rapid clearance of WNV. Various innate immune cells are of importance for the reduction in viral replication and clearance. Activated monocytes isolated from equine peripheral blood show an increased level of NO production and express increased type I IFN levels, leading to the reduction and elimination of WNV infection [172]. In addition, cell counts of microglial cells are greatly increased in the hindbrain and thalamus of experimentally infected equines experiencing neuroinvasive disease, it is expected that they accumulate throughout infection [108]. Furthermore, the presence of WNV antigen in the cytosol of glial cells, macrophages, and neutrophils in the CNS of horses has been observed [151]. Little is known about the role of dendritic cells, NK cells, and the classical complement pathway during equine WNV infection. However, various studies show an involvement of these cells during equine viral infections [173,174,175]. An increase in IRF-7, IFN-α, CXC chemokine ligand 10 (CXCL10), ISG15, and toll-like receptor 3 (TLR3) in the brains of equine patients without WNV neuroinvasion has been observed. Different gene ontology groups including mitogen-activated protein kinase (MAPK), IL-15, IL-22, Janus kinase (JAK)/signal transducer and the activator of the transcription (STAT) signaling, apoptosis pathways, and expression of B and T cell receptors are utilized by equines during the WNV immune response [107]. Similar to humans, in equines an IFN-mediated OAS1 response is activated to attenuate WNV replication. Variation in the OAS1 gene has been associated with variation in susceptibility to WNV infection [176]. A total of six equine SNPs in the promotor region of the OAS1 gene are associated with susceptibility to clinical disease [94].

In equines, the cellular immunity aspect of the adaptive immune system appears to play a role in clearing WNV from the CNS, as infiltration of both CD4+ and CD8+ T cells in approximately equal numbers has been observed in the hindbrain and thalamus of experimentally infected horses [108]. A small subset of peripheral CD4+ T cells can, along with monocytes, be infected by WNV and might play a role in early viral replication before dissemination into the CNS occurs [172]. Although no increase in B cell counts has been observed in the CNS (hindbrain and thalamus) of experimentally infected equines, humoral immunity is important for the clearance of WNV infection [108]. The serological antibody response to WNV lineage 1 and lineage 2 of experimentally infected horses presenting without neuroinvasive disease is similar to each other. WNV-specific IgM antibodies peaked between 8 and 12 days post-infection, after which they steadily declined and were undetectable for four out of six horses at 27 days post-infection [109]. Upon natural infection of horses, an increase in WNV-specific IgM was detected. Of the IgG subtypes, IgG1 is the predominant subtype observed in serum. Both WNV-specific IgM (predominant during primary infection) and IgG1 (predominant during secondary infection) are thought to contribute to virus neutralization during early infection. IgM and IgG1 play an important role in reducing and clearing WNV infection by enhancing the phagocytosis of pathogens through opsonization, their role in the activation of the classical complement pathway, and their virus neutralizing capabilities [177]. During experimental infection, seroconversion and the presence of WNV-specific neutralizing antibodies were observed for all horses by day 12 post-infection and titers increased up to 21 days post-infection [107]. The targeted epitopes of neutralizing antibodies vary between naturally infected horses. For some horses, epitope T332 of domain III (DIII) of the WNV E protein is an important target of the neutralizing antibody pool, while for other horses, the neutralizing antibodies targeting this epitope were undetectable. Therefore, the neutralizing antibody response of equines is thought to vary greatly between individuals. Furthermore, the neutralizing antibody response is thought to be of polyclonal nature and directed to multiple epitopes within and outside of DIII [170]. Passive transfer of WNV-specific maternal antibodies from previously naturally infected mares to their foals has been observed. The WNV-specific antibody titer of the mare is highly correlated (R^2^ = 0.91) to the titer of the foal [178]. In conclusion, equines and humans share similar mechanisms of WNV clearance through both innate and adaptive immunity arms.

## 7. Prophylactic and Therapeutic Strategies

To date, neither vaccine nor therapeutic strategy have been licensed or approved for human use. Some of the recently developed vaccine strategies in the research include replication-competent viral vectors, replication-defective approaches, virus-like particles, nucleic acid vaccines, inactivated vaccines, and synthetic peptides. For yellow fever virus, another virus group in the genus *Orthoflavivirus*, a vaccine was first developed by attenuation of the yellow fever virus in 1937 after serial passage (YF17D). This methodology was commonly used in vaccine development for human and animal diseases [179]. The chimeric approach involving a related *Orthoflavivirus* (YF17D) vector expressing the preM/M-E genes of WNV was successfully used in WNV vaccine research [180]. This chimeric vaccine, developed in the context of YF17D backbone, showed very promising results with good safety in two clinical trials [181]. In addition, WNV-specific monoclonal antibodies that are capable of crossing the blood–brain barrier in laboratory animals are also undergoing clinical testing in humans [182]. Previously, a nanoparticle-based vaccine technology targeting TLR9 was also employed to stimulate T cells and antigen-specific lymphocytes against WNV. Recently, the combination of WNV B and T cell epitopes, universal pan HLA-DR (Pan DR-binding epitope (PADRE); 13 mer) sequence, β-defensin-3 adjuvant (45 mer), and invasion molecule was approached as a one fusion construct towards developing a safe, immunogenic, non-toxic, and highly stable vaccine candidate for WNV disease in humans. Some other prophylactic approaches are being developed, including live attenuated and recombinant vaccines, E protein expression-based deoxyribonucleic acid (DNA) vaccines, NS1 mutant, and non-glycosylated envelop-based attenuated vaccines.

In contrast to the human market, four USDA-licensed vaccines are available for equines. Three USDA-licensed vaccines are whole inactivated virus vaccines, while the fourth one is an *Orthoflavivirus* chimeric inactivated vaccine [183,184]. Furthermore, a therapeutic antibody of equine origin is approved by the USDA [185]. In Europe, three vaccines have been approved for equines, one whole inactivated virus vaccine, one canarypox recombinant vaccine, and one inactivated chimeric yellow fever vaccine [186]. These vaccines can protect horses against WNV infection.

It is important to note that vaccines that are developed for humans undergo more extensive safety testing and are subject to more extensive regulatory requirements in comparison to veterinary vaccines. As a result, the development of human vaccines is much more costly and requires a much bigger financial commitment from companies that are interested in developing a vaccine. The lack of a vaccine for humans is likely a combination of financial and biological limitations. Only two out of six vaccines in clinical trials, as of 2019, were able to induce a strong immune response after a single dose. Both of these were live attenuated vaccines which are more difficult to obtain licensing for than other vaccine platforms due to safety concerns [187]. For the successful development of a WNV vaccine for humans, several factors need to be researched in detail. For example, the probability of the vaccine to induce antibody-dependent disease enhancement (ADE) due to pre-existing antibodies produced in response to other *Orthoflaviviruses* should be determined and, if applicable, should be avoided [188]. Vaccination may also lead to more severe symptoms during secondary infections of other viral infections, which should be minimized as well [189]. While it is not likely that antibodies to the DIII domain cause ADE for other viruses, it should be further explored through the introduction of mutations in the DIII of the E protein or alternative methods to diminish the likelihood of antibodies binding to other *Orthoflaviviruses* [190]. Ultimately, the vaccine should be able to induce a strong adaptive immune response that is safe for its host and is able to restrict WNV infection [191].

## 8. Successful Vaccination of Equines against WNV and Potential Lessons for Human Vaccine Development

As described before, multiple successful vaccines using various platforms are available for equines, yet to date, no vaccine is available for human use. In 2003, the first WNV vaccine was approved by the USDA which was an inactivated whole virus vaccine. In the years afterward, the number of equine cases decreased greatly, unlike human cases (Table 1) [192]. Since then, various additional vaccine platforms have been researched and licensed by the USDA and European agencies. In vaccination experiments, one of the inactivated whole virus vaccines, West Nile Innovator, showed an ability to induce antigen-specific antibody responses, as well as activation of CD4+ and CD8+ lymphocytes in response to three vaccinations with 3-week intervals between them. Following vaccination, IFN-γ and IL-4 were elevated in an antigen-specific manner in CD4+ and CD8+ peripheral blood mononuclear cells (PBMCs), indicating Th1 involvement. Furthermore, cellular proliferation of CD4+ and CD8+ lymphocytes after cultivation with WNV antigen was measured and showed an increased proliferation of these cells for, respectively, 100 and 120 days after vaccination. An increased proliferative response was also measured for B lymphocytes after two doses of the vaccine which was maintained for 100 days. Measurement of serum Ig levels showed increases in IgM, IgA, IgGa, IgGb, IgGc, and IgG(T) following the three doses of vaccination which contained neutralizing capabilities (>1:100) and remained elevated for the duration of the experiment, which was six months. However, the unvaccinated horses also developed a neutralizing antibody response following natural exposure to WNV [193]. Another inactivated whole vaccine developed by Fort Dodge Animal Health was shown to be safe and effective in clinical trials. In this study, horses were vaccinated twice using a 14 day interval between each dosage. A total of 12 months after the second dosage, 19 vaccinated horses and 11 control horses were experimentally infected with WNV. Out of the 11 unvaccinated control horses, 9 (81.8%) developed viremia post challenge. However, vaccination protected 18 of the 19 vaccinated horses from viremia development; therefore, the vaccine is expected to be 94% effective. The follow-up safety study involving 648 horses showed no adverse effects due to vaccination for 96% of the horses and only a small number of horses developed mild and transient injection site reactions. This study also included 32 pregnant mares that showed no adverse reactions; therefore, this vaccine is also considered safe to use in pregnant mares [194]. Vaccination of foals at the age of 90 days using three doses of a multivalent WNV vaccine has demonstrated an ability to raise immune response despite the common practice of only vaccinating foals over six months of age due to maternal antibody interference. CD4+ and CD8+ lymphocytes showed antigen-specific expression of IL-4, IFN-γ, and granzyme B 30 days after initial vaccination, and a booster at 11 months of age increased the antigen-specific IgG response, indicating good memory immune responses [195]. A study including 240 horses examined the effect of multivalent WNV vaccination. Although all vaccines stimulated immune responses higher than the unvaccinated and seronegative controls, the inclusion of equine encephalitis viruses or tetanus toxoids decreased the neutralizing antibody response to WNV [196]. Upon experimental infection of four horses who were previously negative for neutralizing antibodies, neutralizing antibody titers reached >1:320, 1:20, 1:160, and 1:80, respectively, for each horse, it is unclear if this would provide sufficient protection for reinfection or how long such titers last [82]. Experimental infection of 28 horses after intramuscular vaccination consisting of two doses at 5-week intervals using a recombinant canarypox virus vaccine which carried the prM and E genes of WNV was shown to be protective against WNV infection. Neither the vaccinated nor unvaccinated horses developed clinical signs; however, 80% of unvaccinated horses became infected as shown by viremia levels. One horse in the medium-dose vaccination group failed to develop neutralizing antibodies and developed viremia. All other vaccinated horses did not develop viremia and had sufficient neutralizing antibodies as a result of vaccination [197]. Although annual boosters are required, equine WNV vaccination has shown to be protective and decreased equine cases after the introduction of vaccination. Based on information about equine vaccination, human vaccines should aim to acquire similar immune response properties. Furthermore, since vaccination of foals has been shown to be effective, vaccination of children should be accomplishable. WNV vaccination for humans should try to avoid the use of multivalent vaccines, since in horses this has been shown to decrease the effectivity of certain pathogen combinations. The development of a successful vaccine for humans will likely lead to a reduction in cases and deaths as seen in equines.

## 9. Equines as a Model Animal for WNV Infection in Humans

**Figure 2:**
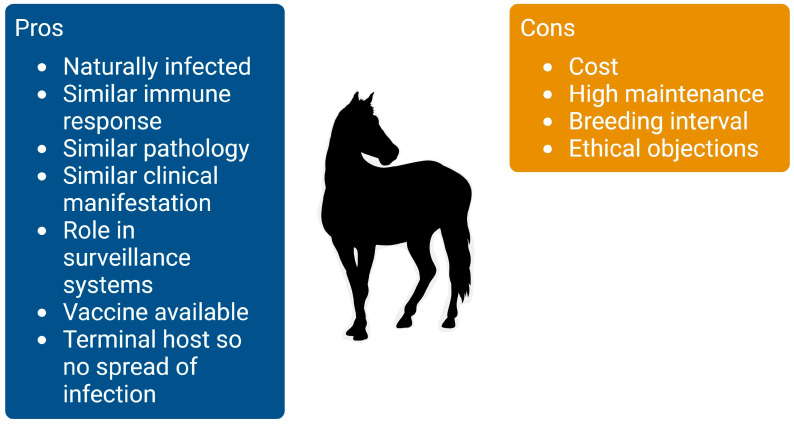
Pros and cons of using equines as an animal model for WNV infection in humans.

Various animal models including mice, hamsters, rabbits, and non-human primates have been used to study the infection kinetics and pathogenesis of WNV infection which has recently been reviewed by Byas and Ebel [149]. However, current models have limitations; for example, challenge of outbred wild-type mice is possible using intraperitoneal infection [198]. However, mice with a mutation in their OAS1b gene are used more commonly. Furthermore, adult rats are resistant to WNV infection and only neonates can be used as a model. Non-human primates have been used in the past but are used less frequently due to financial costs and ethical objections [149]. Although these animal models have provided significant insights into the study of pathogenesis, we think that equines would serve as a better biomedical model for the evaluation of potential human vaccines. Equines can provide valuable information as a model species for a number of human infectious diseases such as HIV, influenza, and herpesvirus [199]. Because of the similar clinical manifestation, pathogenesis, and immune responses of equine WNV infection, equines are an excellent candidate as a model animal for human WNV infection as well. Although equines would be a great model system for WNV in humans there are several limitations (Figure 2). For example, breeding and management is more extensive compared to smaller animal models, one would need a bigger research facility to maintain a herd of horses compared to a colony of mice. Furthermore, because of their higher maintenance and facility requirements equines are more expensive to maintain; however, costs can be reduced by using smaller breeds such as Shetland ponies for example. Equines also have a longer generation interval compared to small animal models; it takes about 2 years for a horse to become skeletally mature while this process only takes 26 weeks for a mouse. As the species most frequently and significantly affected by WNV infection other than birds and humans, equines have been used in vaccine studies for WNV [200,201]. Despite these limitations, equines will be an excellent model because of their susceptibility to (natural) infection, clinical manifestation, and role in surveillance systems. A study conducted on equines for the WNV reported low-level viremia that is intermittently detectable and feeding mosquitoes failed to transmit the disease [82]. Several experimental studies used horses as dead-end hosts for vaccine efficacy trials and suggested that mosquito feeding or subcutaneous inoculation rarely result in overt clinical disease [194,197,202]. Moreover, an effective vaccine is available for equines which can be used to study protective immune parameters. Because equines, like humans, are dead-end hosts, the chances of the infection spreading following experimental infection is limited in contrast to birds. This makes them a more appropriate model for human studies in comparison to any other experimental models [203].

## 10. One Health Perspective of WNV

WNV has a complex transmission cycle involving human, veterinary, and environmental interfaces. Being a zoonotic disease that involves various hosts in its replication and transmission cycles makes WNV a suitable candidate for a One Health approach for research and surveillance strategies (Figure 1). Approximately 64% of human pathogens are zoonotic and 5% of the total number of human pathogens are viruses [204]. Recently emerged zoonotic pathogens are responsible for 26% of the infectious disease burden in developing countries and 0.7% in developed countries [205]. Although the life cycle of WNV revolves around mosquitoes and birds with humans and other animals such as equines as dead-end hosts, environmental factors can influence greatly how these hosts are connected to one another and can contribute to changes in the prevalence of the disease. For example, alterations in climate conditions such as temperature and precipitation are driving forces of the adaptation in the range of WNV-susceptible mosquitoes leading to an altered probability of the disease spreading to other hosts in the affected geographical regions [206]. Circumstances such as the presence of wetlands, packed urbanization, industrialization, and agricultural activities, leading to an increase in stagnant water, provide a suitable environment for mosquitoes to breed and amplify WNV. Similarly, woodlands and bushy areas offer a suitable place for vectors and reservoirs of WNV, which can lead to spread of WNV to equines when they are situated near these habitats [207,208,209]. Furthermore, the natural migration of birds, the trade of these birds by humans, globalization, cultural activities, traditions, and the general behaviors of humans can greatly impact the prevalence of WNV outbreaks in humans and equines in certain regions [210]. Thus, WNV utilizes an intricate and connected network of hosts which are all related to one another. Circumstances affecting WNV infection in one host can trigger a chain reaction that influences other hosts as well, making WNV an ideal candidate for One Health approaches.

Surveillance programs for one host can provide insights into current WNV prevalence in general and can be used as an indicator of potential outbreaks for other hosts. The One Health approach to disease surveillance is considered a cost-effective approach for combating the spread of zoonotic viral diseases [211]. Various health organizations have implemented a One Health approach with success. For example, the implementation of the One Health approach for monitoring WNV infection of the first identified human case of West Nile Neuroinvasive Disease (WNND) in Sicily lead to the discovery of seropositivity in horses and dogs on farms surrounding the patient’s residence [212]. Furthermore, the European Union successfully utilizes the One Health approach for surveillance of both human and veterinary cases; all cases are reported to ECDC. Based on these data, a correlation between the increase and decrease in equine cases and the related increase and decrease in human cases has been shown [213,214]. Lastly, the USDA uses its National Animal Health Surveillance System (NAHSS) to collect data related to WNV infection in equines and collaborates with the Centers for Disease Control and Prevention (CDC) regarding reported human cases [215]. Reports of equine cases could potentially provide an early warning of the same WNV transmission in human populations [11,216,217]. The sectoral approach used so far has been shown to be insufficient for combating WNV outbreaks and the development of a suitable vaccine for humans. A One Health approach leading to a better understanding of the host factors involved in the spread of disease, pathogenesis, and immune-related parameters could contribute to controlling the spread of WNV infections and the development of a suitable vaccine for humans.

## 11. Conclusions and Future Directions

WNV was first identified in humans in 1937, and global outbreaks in dead-end hosts such as humans and equines have been reported ever since. In 1999, WNV was first observed in the USA, after which the number of human cases and deaths remained high due to the absence of an effective vaccine. However, due to the introduction of an equine vaccine, equine cases and deaths have dramatically decreased. The development of the human vaccine could greatly benefit from equines as a model species for WNV infection due to the similarities between equine and human clinical manifestation, pathogenesis, and immune responses to WNV infection. Since most factors influencing WNVs’ life cycle have an influence on the system as a whole and not just one species, it is highly recommended to use a One Health approach for future research. More communication and collaboration are needed between clinicians, animal health advisors, and entomologists to reach their collective goal of producing better preventative strategies, such as an effective vaccine for humans, to decrease the number of human cases and deaths.

## Figures and Tables

**Table 1:** Equine and human cases, and human deaths in the United States of America (USA) from 1999 to 2022 [32,36,37].

Year	Equine Cases	Human Cases	Human Deaths
1999	25	62	7
2000	60	21	2
2001	738	66	10
2002	15,257	4156	284
2003	5181	9862	264
2004	1406	2539	100
2005	1088	3000	119
2006	1086	4269	177
2007	468	3630	124
2008	179	1356	44
2009	276	720	32
2010	125	1021	57
2011	87	712	43
2012	627	5674	286
2013	377	2469	119
2014	141	2205	97
2015	225	2175	146
2016	380	2149	106
2017	307	2097	146
2018	493	2647	167
2019	90	971	60
2020	71	731	66
2021	220	2911	227
2022	NA ^1^	1126	90

^1^ NA—no data available for equines in 2022.

## Data Availability

No new data were created or analyzed in this study.
